# Making the COVID-19 crisis a real opportunity for environmental sustainability

**DOI:** 10.1007/s11625-021-01003-z

**Published:** 2021-07-12

**Authors:** Paul Lehmann, Mariana Madruga de Brito, Erik Gawel, Matthias Groß, Annegret Haase, Robert Lepenies, Danny Otto, Johannes Schiller, Sebastian Strunz, Daniela Thrän

**Affiliations:** 1 Department of Economics, Helmholtz Centre for Environmental Research—UFZ, Permoserstr. 15, 04318 Leipzig, Germany; 2 Faculty of Faculty of Economics and Management Science, University of Leipzig, Grimmaische Straße 12, 04109 Leipzig, Germany; 3 Department of Urban and Environmental Sociology, Helmholtz Centre for Environmental Research—UFZ, Permoserstr. 15, 04318 Leipzig, Germany; 4 Institute of Sociology, Friedrich-Schiller-University Jena, Carl-Zeiß-Straße 3, 07743 Jena, Germany; 5 Department of Environmental Politics, Helmholtz Centre for Environmental Research—UFZ, Permoserstr. 15, 04318 Leipzig, Germany; 6 Department of Bioenergy, Helmholtz Centre for Environmental Research—UFZ, Permoserstr. 15, 04318 Leipzig, Germany

**Keywords:** COVID-19, Green recovery, Pandemic, Sustainability transitions

## Abstract

An optimistic narrative has gained momentum during the first year of the pandemic: the COVID-19 crisis may have opened a window of opportunity to “rebuild better”, to spur societal transitions towards environmental sustainability. In this comment, we review first evidence of individual and political changes made so far. Findings suggest that economies worldwide are not yet building back better. Against this background, we argue that a naïve opportunity narrative may even impair the progress of transitions towards environmental sustainability because it may render green recovery measures ineffective, costly, or infeasible. Based on these observations, we derive conditions for green recovery policies to succeed. They should consist of a policy mix combining well-targeted green subsidies with initiatives to price emissions and scrap environmentally harmful subsidies. Moreover, green recovery policies must be embedded into a narrative that avoids trading off environmental sustainability with other domains of sustainability—and rather highlights respective synergies that can be realized when recovering from the COVID-19 crisis.

## Introduction

The COVID-19 crisis—produced by the pandemic and the measures taken to mitigate it—has led to severe economic and social impacts. Nonetheless, an optimistic narrative has gained momentum during the first year of the pandemic: the COVID-19 crisis may have opened a window of opportunity to “rebuild better”, to spur societal transitions towards environmental sustainability. Numerous authors have raised this expectation (e.g., Bodenheimer and Leidenberger 2020; Cloete 2020; Rosenbloom and Markard 2020; Markard and Rosenbloom 2020; Sarkis et al. 2020; Steffen et al. 2020). Most prominently, the opportunity narrative was put forward at the World Economic Forum’s “The Great Reset” event by the Prince of Wales: “We have a golden opportunity to seize something good from this crisis — its unprecedented shockwaves may well make people more receptive to big visions of change” (Taylor 2020).

The opportunity narrative goes that the COVID-19 crisis may trigger societal change through two channels: First, the shock may lead to individuals and companies reviewing their behavioral routines and business models (“individual change”). Individuals may be triggered to contemplate about their lifestyles due to the personal experience of a global crisis and the timeout produced by lockdowns. Companies may be forced to develop more crisis-proof business models in response to the sudden interruption of global supply chains. Second, the crisis may also allow for changes of policies and institutions which would not have been otherwise feasible (“political change”). The COVID-19 crisis has made trillions of public money available for expenditure at short notice—something which was unthinkable before the crisis. This observation may nourish confidence that governments are actually able to act and respond to global crises if there is sufficient political will.

Consequently, the COVID-19 crisis may be perceived as an opportunity to facilitate the transition towards more sustainable modes of consumption and production. The crisis might lead to the societal change which would be necessary to mitigate adverse environmental change. But how likely is such a transition to occur in the aftermath of the COVID-19 crisis? In this comment, we will stress that the COVID-19 crisis is not an opportunity for societal change per se—and if so, it is unclear whether individual and political changes induced by the crisis will actually lead to more environmental sustainability in the long run. Thus, societal changes induced by the COVID-19 crisis do not necessarily mitigate adverse environmental change. Against this background, we will argue that a naïve opportunity narrative may even impair the progress of transitions towards environmental sustainability because it may render green recovery measures ineffective, costly, or infeasible. Based on these observations, we will derive conditions for green recovery policies to succeed.

The notion of a “window of opportunity” goes back, inter alia, to Kingdon (1984), who primarily looked at drivers of political change. He argued that a window of opportunity for political change may open up if (1) there is high attention to a problem, (2) a feasible solution is available, and (3) policy-makers have the motive and opportunity to accept it. How crises may (or may not) trigger political (or, more generally, institutional) change has been extensively discussed by the social science literature of institutional analyses and transition studies (for an overview, see Haase et al. 2018). In addition, behavioral scientists have tried to understand how and when crises may lead to individual change (for an overview, see Schäfer et al. 2012). In our comment, we combine insights from these literature strands to derive hypotheses regarding how the COVID-19 crises may affect societal change. While there is much talk about possible opportunities created by the COVID-19 crisis in the literature, it usually remains fuzzy what exactly constitutes an opportunity, and what does not (Bodenheimer and Leidenberger 2020; Markard and Rosenbloom 2020; Sarkis et al. 2020). Our comment aims to add to this literature by specifying the conditions that are necessary for the COVID-19 crisis to actually spur the transition towards environmental sustainability. In addition, we summarize first empirical evidence underpinning or challenging the theoretical hypotheses regarding the impact of the COVID-19 crisis on societal change.

## The difficult notion of “opportunity”

We believe the notion of “opportunity” needs to be used very carefully. Whether or not an opportunity for individual and political change arises out of the COVID-19 crisis depends a lot on how vulnerable individuals and societies are. The COVID-19 crisis has a disproportionately negative effect on the most vulnerable groups. For instance, poor households are most affected by job and earnings losses due to lockdowns (Asayama et al. 2020). In Canada, for example, employment losses among low-wage employees, between February and April 2020, were more than twice as high as the losses among all paid employees (Galasso and Foucault 2020). The impacts of the COVID-19 crisis on the poor are even more disproportionate in developing countries, where living conditions of the poor are particularly precarious (increasing the risk of infection), and access to social services and relief is limited (aggravating the economic consequences of lockdowns) (Asayama et al. 2020; Orendain and Djalante 2021). The World Bank (2021) estimates that the COVID-19 lockdowns have pushed around 100 million people into extreme poverty worldwide in 2020 alone (more than half of them in South Asia). As a consequence, preliminary evidence suggests that the COVID-19 pandemic has undermined progress in 12 of the 17 sustainable development goals (SDGs), such as no poverty (SDG1), zero hunger (SDG2), or reduced inequalities (SDG 10) (Leal Filho et al. 2020; Sachs et al., 2020). For those vulnerable groups which are hardest hit, the COVID-19 crisis hardly constitutes an opportunity for societal change, but rather an existential threat, dominated by concerns over day-to-day survival. Any environmental benefits resulting from these hardships—such as reduced consumption or mobility—can hardly be considered as desirable in terms of sustainability. They simply buy environmental sustainability at the cost of social sustainability. Moreover, recent research indicates that such changes will likely be temporary, and reversed once the crisis is over (Freire-Gonzáles and Vivanco 2020; Li and Li 2021).

Similarly, crises may disproportionally affect some companies. Green niche innovators may be particularly vulnerable, as these tend to have fewer financial reserves than incumbents (Geels 2013). In the US, lockdowns led to a loss of over half a million clean energy jobs in the first months of the COVID-19 pandemic (Blackmon 2020). Again, impacts may be particularly adverse in developing countries where the crisis has resulted in a depreciation of currencies and higher borrowing costs. This particularly impairs investments in renewable energy technologies due to their high capital intensity (Quitzow et al. 2021). Consequently, green investments necessary for sustainability transitions are being delayed in many countries (Döttling and Kim 2021).

Therefore, it may be flawed to homogenize any changes induced by the COVID-19 crisis as “opportunities”. A legitimate opportunity for societal change spurring the transition to environmental sustainability only arises when society is able to satisfy its basic needs.

## Ambiguous sustainability effects of individual change

Even if the COVID-19 crisis creates an opportunity for individual change, it will only promote environmental sustainability under certain conditions. The shift in mobility and working practices in times of COVID-19 provides an example. Digitalization has accelerated in several industries, and many people are now working remotely (Sarkis et al. 2020), also in science (Leal Filho et al. 2021). Using the example of one of the biggest European Political Science conferences, Jäckle (2021) estimates that organizing the conference online in 2020 has reduced the carbon footprint of participants by 99%, compared to conferences held in-person.

Yet, the respective environmental gains may be thwarted at least partly by rebound effects which are substantial for the intensified use of information and communication technologies (Freire-González and Vivanco 2020). Moreover, those people still commuting are increasingly switching from public transport to individual cars to avoid the risk of infection. A ten-country survey by Barbieri et al. (2021) found that, while mobility was reduced across all transport modes, the decline was most pronounced for public transport and least pronounced for cars. Similarly, several other studies show that the share of car usage in the modal split has increased in several countries (Bucsky 2020; Eisenmann et al. 2021; Molloy et al. 2021; Przybylowski et al. 2021).

Moreover, it is ex-ante unclear how enduring any such individual changes in behavior and business models are. Take again the example of adjusted mobility and working practices. On the one hand, positive learning experiences made with remote-working may help to change practices permanently. This effect is likely to increase with the duration over which people are compelled to change social practices (Boons et al. 2020). The longer new social practices are adopted, the more likely they are to become the new “normal”. On the other hand, behavioral changes may rebound at least partly once lockdowns are relaxed and the immediate fear of infection vanishes (Schäfer et al. 2012). Despite the shock of the crisis, humans may be tempted to return to established social practices as soon as they are allowed to. This expectation may be underpinned by a survey carried out in the Netherlands by de Haas et al. (2020) during the COVID-19 crisis. 80% of the respondents did not plan to change their travel mode permanently. Only 27% of the surveyed home-workers expected to work from home more frequently in the future. And if individual changes do turn out to last, this may apply to both environmentally beneficial as well as environmentally harmful changes. First surveys suggest that COVID-19 may leave a permanent shade on sustainable modes of transportation like public transport and increase car dependence (de Haas et al. 2020; Zhang et al. 2021).

Recent data on the development of global CO_2_ emission in 2020 nourish the expectation that certain environmentally beneficial individual changes may not last for long. On an annual basis, global CO_2_ emissions declined by up to 7% in 2020 (IEA 2021; Le Quéré et al. 2021). This reduction was primarily due to short-term changes in individual behavior and production, which resulted from the pandemic as well as the public measures taken to mitigate it. Restrictions on local and international mobility in the transport sector accounted for 50% of these reductions. However, CO_2_ emissions are quickly bouncing back. Global December emissions were already 2% higher than they had been in the same month a year earlier. In China—one of the first countries to relax COVID-19 lockdowns—emissions have been back above 2019 levels already from April 2020 on. This suggests that the individual changes induced by COVID-19-restrictions do not seem to last beyond lockdowns. Hence, a well-known pattern from previous crises—like the oil crises in the 1970s or the 2007–08 global financial crisis—seems to come to light again: Emissions growth has picked up quickly after every crisis so far, if not immediately, then within a few years (Hanna et al. 2020; Le Quéré et al. 2021).

## Ambiguous sustainability effects of political change

The recently observed rebound in emissions illustrates how important political change is for sustainability transitions to be spurred in times of COVID-19. The pandemic crisis has certainly opened a window of opportunity for political change as there is broad public support for quick and strong political action. The enormous amount of fiscal stimulus programs issued by governments across the globe—currently some US$ 14.9 trillion have been announced (Vivid Economics 2021)—would not have been politically feasible without the COVID-19 pandemic. If this money is spent on green, low-carbon investments, stimulus programs may facilitate the transition towards environmental sustainability. However, whether this actually happens in times of crisis depends on a variety of factors, such as the severity and type of crisis, the public and media framing, socio-economic capacities, and interests to “rebuild better” (Geels 2013; Haase et al. 2018; Hay 1999). In principle, crises may also hinder or delay sustainability transitions as unemployment and economic problems often dominate immediate political concerns and debate (Ashford et al., 2012). This may be the case particularly if crises have a disproportionately negative impact on vulnerable groups and aggravate existing inequalities—as has been pointed out for the COVID-19 crisis above. Löschel et al. (2020) and Zhang et al. (2020) find, for example, that political support for environmental policies has declined in Germany and China during the COVID-19 pandemic. In contrast, Evensen et al. (2021) find little evidence for diminishing climate change concerns in the UK.

**Fig. 1:**
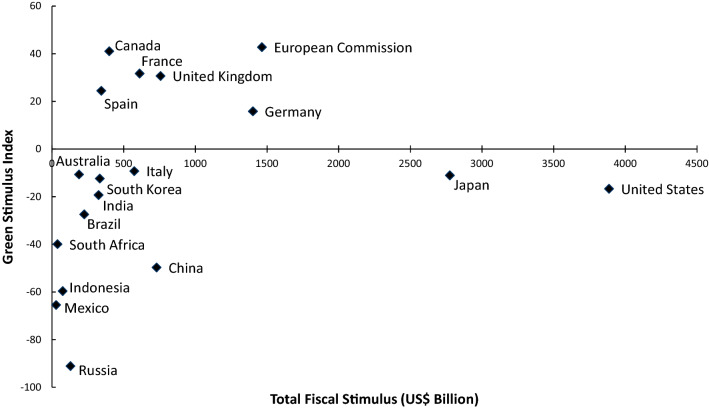
Total fiscal stimulus adopted by selected countries and its greenness (Source: Vivid Economics 2021)

Existing fiscal stimulus programs show a very ambiguous picture in terms of their greenness (see Fig. 1). Vivid Economics (2021) estimates that only 40% of the stimulus dedicated to sectors with direct impacts on emissions (agriculture, industry, waste, energy, and transport) are green. Stimuli in 15 of the G20 countries have a net negative environmental impact. UNEP (2021) even finds that only 18% of the long-term recovery-types measures are targeted at green recovery initiatives.

Figure 1 illustrates that the direction of political responses seems to depend crucially on the general political will and interest to rebuild more sustainably. The EU (particularly its Next Generation EU package) and several of its Member States have been fairly ambitious in greening recovery policies, also because they had already embarked on a green new deal track before the COVID-19 crisis (Dupont et al. 2020; Smith 2020; Vivid Economics 2021). In contrast, emerging economies like Russia, Mexico, and Indonesia, which strongly depend on fossil-fuel exports, have announced environmentally harmful stimuli which directly support long-lived investments in oil, gas, and coal industries (Quitzow et al. 2021; Vivid Economics 2021). Similarly, major producers of agricultural commodities—like Brazil or Indonesia—have loosened permitting procedures for timber producers, which is likely to foster deforestation. Recovery efforts of major economies—like the US, China, India, or Japan—have ambiguous (but on average still slightly negative) environmental impacts. Yet, at least those stimuli additionally implemented in the last months have shifted significant amounts of money to renewable energies and low-carbon mobility technologies (Gosens and Jotzo 2020; Smith 2020; Vivid Economics 2021).

Figure 1 also highlights that the strength of political responses—and thus the actual opportunity for political change—hinges on the capacities of the respective countries. Notably, emerging economies like India, Russia, or Indonesia have issued much smaller stimuli programs than advanced economies like the US or the EU (Vivid Economics 2021). High interest rates and existing debt constraints have limited the recovery efforts of emerging and developing economies (UNEP 2021).

Against these observations, UNEP (2021) concludes that economies worldwide are not yet building back better. Moreover, the political action taken in response to the COVID-19 crisis may deepen the gap between leaders and laggards in the transition towards environmental sustainability (Quitzow et al. 2021).

## Risks of a naïve “opportunity” narrative

The first evidence on the extent and direction of individual and political changes suggests that expectations of a momentum for sustainability transitions being created by the COVID-19 crisis have been overly optimistic. The societal change induced by the crisis has not necessarily facilitated the mitigation of adverse environmental change. An opportunity for environmental sustainability only arises if the crisis induces sufficient and enduring individual and political change, and if this change is actually targeted at environmentally more sustainable choices. Anyone ignoring this conditionality runs the risk of being taken in by a naïve opportunity narrative. Importantly, such naivety may impair the progress of transitions towards environmental sustainability, rather than facilitating it.

First, a naïve “opportunity” narrative may lead to environmental sustainability transitions being ineffective. It may make decision-makers rely on individual changes towards more environmental sustainability observed during the COVID-19 crisis—like advanced digitalization. Yet, it may oversee that these are not necessarily enduring, or may be more than compensated by rebound effects, unless supported by strategic policy intervention (Freire-González and Vivanco 2020).

Second, a naïve opportunity narrative may lead to environmental sustainability transitions being guided by policies with inappropriate priorities. Policy decisions are typically driven by issue-attention cycles (Downs 1972): Catastrophic events, like the COVID-19 pandemic, tend to lead to short-term changes in political priorities. Consequently, only those sustainability issues may show up on political agendas now for which a direct nexus to the COVID-19 crisis can be established, e.g., health and biodiversity conservation. Other important challenges may lose political momentum. While it is generally important to reap co-benefits between pandemic control and other societal challenges where possible, it would certainly be flawed to focus political efforts solely on measures providing such co-benefits. First evidence based on Google Trends data finds that public awareness regarding natural resources, like green spaces or biodiversity, has increased during the first months of the pandemic (Rousseau and Deschacht 2020). In contrast, public awareness of other issues like climate change or circular economy remained unchanged. Lyytimäki et al. (2020) even find a substantial drop in media coverage regarding climate change after the pandemic emerged.

Third, a naïve opportunity narrative may lead to environmental sustainability transitions being guided by policies with inappropriate measures. The expectation of huge amounts of public recovery money being distributed at short notice opens up a “pork barrel” to lobbyists who pursue specific technological interests (Helm 2010). Green expenditures require governments to specify which technologies to support at what rate. Yet, governments may be imperfectly informed about the actual greenness and costs of technologies. This may allow better informed lobbyists to seek rents. Moreover, the short-term abundance of public recovery funds impedes competition between interest groups, which could otherwise limit rent-seeking behaviour (Lehmann and Söderholm 2018). Hence, the “opportunity” narrative may be used to justify any type of policy interventions—preferably those involving government expenditures—as long as they can be labelled as green. The actual (cost-) effectiveness of these measures may be ignored, leading to a waste of public resources. In the worst case, the spending of recovery funds would turn out to be ineffective environmentally, while at the same time opening up for windfall profits and fraud. In Italy, for example, there are concerns that a substantial part of recovery funds might be captured by organized crime (Follain and Migliaccio 2021).

Finally, a naïve “opportunity” narrative may compromise sustainability transitions in general. It may create the impression that any hardship experienced during the COVID-19 crisis is an acceptable and necessary price for environmental sustainability transitions to succeed. This would violate the idea of a “just transition”, i.e., a managed shift towards low-carbon systems which also prioritizes secure, family sustaining jobs and healthy communities (Henry et al. 2020). Moreover, such a narrative may lead to people associating sustainability transitions with an excessive restriction of individual rights (as during the lockdown), or with a general economic downturn. This would make it even more difficult to win political majorities for sustainability transitions.

## Smart policy choices to deliver a green recovery

To avoid the risks created by a naïve “opportunity” narrative, and to actually deliver on a green recovery, political choices need to be guided by clear criteria. First of all, the window of opportunity created by the COVID-19 crisis can only be used effectively to spur the transition to environmental sustainability if individual change combines with political change directed towards sustainability. Only maintaining and strengthening environmental regulation will safeguard that individuals switch to sustainable modes of production and consumption. Consequently, it has been a success that the European Union has withstood pressures from some Member States and industry interest groups to suspend the EU emissions trading scheme, to postpone the tightening of CO_2_ emissions standards for cars, or to abandon the European green deal as a whole in response to the COVID-19 crisis (Dupont et al. 2020; Elkerbout et al. 2020). In turn, the reversal of environmental regulation and the implementation of new subsidies to fossil fuels—as, for example, observed last year in Russia, Brazil, or Indonesia (Vivid Economics 2021)—certainly impedes any opportunity to rebuild the economy more sustainably.

It is reasonable to complement existing environmental regulations by green recovery programs. However, these must not be arbitrary and should go beyond green subsidies. Programs also need to price environmental externalities, dismantle environmentally harmful subsidies, and provide information necessary to take sustainable investments (as by the EU taxonomy for sustainable finance). Otherwise, green subsidies may turn out to be expensive and ineffective policy tools for sustainability transitions (see, for example, the discussion of renewable energy subsidies in Kalkuhl et al. 2013 and Palmer and Burtraw 2005). Experiences made with green stimulus packages implemented after the 2007–08 global financial crisis confirm this argument (Barbier 2020). While they promoted the deployment of green technologies, like energy-efficient appliances or renewable energies, they hardly led to a reduction in CO_2_ emissions. Reductions in CO_2_ emissions from using green technologies were more than offset by increasing energy use from fossil fuels. The main reason for this development was the absence of effective carbon pricing which could have curtailed the use of fossil fuels. This is still a relevant shortcoming. According to the World Bank (2020), currently only 22% of all greenhouse gas emissions are subject to carbon pricing. Particularly emissions in the transport, building, and agriculture sectors are often not subject to carbon pricing. More generally, carbon pricing schemes do not exist in most developing and emerging economies. So, particularly in these sectors and countries, green recovery programs may fail in improving environmental sustainability if they do not include carbon pricing initiatives. Carbon pricing reforms may also contribute, at least partly, to funding (green) recovery programs (Stern et al. 2020).

Green subsidies should focus on measures for which public support was justified already before the COVID-19 crisis, e.g., to address technology market failures existing next to the CO_2_ externality (Bennear and Stavins 2005; Lehmann 2012). Moreover, subsidies should be targeted at measures that have the highest priority for environmental sustainability, and for which rational concepts ready to implement have been drafted already (Gawel and Lehmann 2020). One such priority area is the transport sector. This is clearly lagging behind in terms of decarbonization—and this deficit may be even aggravated now as the COVID-19 crisis may lead to an additional shift from public transport to individual mobility. Moreover, carbon pricing alone will not suffice to decarbonize this sector. Technology choices are strongly path-dependent on the historically developed infrastructure for individual mobility based on fossil fuels (Briggs et al. 2015; Low and Astle 2009). Consequently, public support is warranted to roll out new infrastructures for low-carbon mobility options, like charging stations for e-mobility, public transport or, more generally, walking- and cycling-friendly cities. In contrast, it is at least unclear how helpful direct subsidies to purchasing low-carbon vehicles are, particularly if carbon pricing and complementary infrastructure are insufficient. Previous “Cash for Clunkers” programs warn as an example of a misguided recovery measure. These programs were introduced in many countries after the 2007–08 global financial crisis and provided financial incentives to trade old, less fuel-efficient cars for new, more efficient ones. Yet, the performance of these programs has been very mixed regarding both economic and environmental stimulus effects (Grigolon et al. 2016; Li et al. 2013; Mian and Sufi 2012). Thus, these examples illustrate that green recovery programs should combine green carrots and sticks, and they should be picky on green carrots.

More generally, green recovery efforts must build on a narrative that avoids selling occasional and short-term windfall profits from an emergency as a gain for sustainability. A successful narrative tells a winning story of a long-lasting transition towards environmental sustainability and also pays sufficient attention to potential trade-offs with the economic and social domain of sustainability (Asayama et al. 2020). Ideally, such a narrative also stresses the potential synergies which may be created between environmental sustainability and other sustainability domains if green recovery policies are properly designed. Supporting green investments—for example, those related to clean energy infrastructures or insulation retrofits—may also pay off in terms of economic and social development. Typically, green investments have large job multipliers because they are labor-intensive and less likely to be offshored to imports (Hepburn et al. 2020; Stern et al. 2020; UNEP 2021). Targeting public funds at green investments may also help to compensate for the fact that green industries may be particularly affected by the COVID-19 crisis. What is more, particularly in rural areas of developing countries, the publicly funded roll-out of renewable energies and sustainable agriculture may also help mitigating water and energy poverty (Barbier and Burgess 2020).

In sum, the disruptive shocks produced by the COVID-19 crisis, the enormous amounts of public recovery money provided, and more generally the broad public support for political action to mitigate the crisis have certainly opened a window of opportunity for societal change. Yet, the size of this window depends on the specific regional context in which individual and societal decisions are taken. In any case, the crisis has shown that governments can take rapid and drastic measures to respond to crises—but only if a sense of urgency and political will are strong enough. Moreover, this only turns into a real opportunity for mitigating adverse environmental change if individual changes are combined with political changes that rest on smart and targeted recovery policies. These policies need to be embedded in a truly sustainable narrative for a recovery that integrates environmental, economic and social concerns. More specifically, recovery efforts should be designed to be part of the necessary long-term sustainability transitions. In other words, COVID-19 must be actively made an opportunity for sustainability by policy-makers, companies, scientists, and civil society.
